# The role of information provision in economic evaluations of non-invasive prenatal testing: a systematic review

**DOI:** 10.1007/s10198-019-01082-x

**Published:** 2019-06-22

**Authors:** Nikita M. John, Stuart J. Wright, Sean P. Gavan, Caroline M. Vass

**Affiliations:** Manchester Centre for Health Economics, Division of Population Health, Health Services Research and Primary Care, The University of Manchester, Jean McFarlane Building, Oxford Road, Manchester, M13 9PL UK

**Keywords:** Aneuploidy, Down’s syndrome, NIPT, Prenatal testing, Information, Cost effectiveness, I19, D61

## Abstract

**Background:**

Technological progress has led to changes in the antenatal screening programmes, most significantly the introduction of non-invasive prenatal testing (NIPT). The availability of a new type of testing changes the type of information that the parent(s) require before, during and after screening to mitigate anxiety about the testing process and results.

**Objectives:**

To identify the extent to which economic evaluations of NIPT have accounted for the need to provide information alongside testing and the associated costs and health outcomes of information provision.

**Methods:**

A systematic review of economic evaluations of NIPTs (up to February 2018) was conducted. Medline, Embase, CINAHL and PsychINFO were searched using an electronic search strategy combining a published economic search filter (from NHS economic evaluations database) with terms related to NIPT and screening-related technologies. Data were extracted using the Consolidated Health Economic Evaluation Reporting Standards framework and the results were summarised as part of a narrative synthesis.

**Results:**

A total of 12 economic evaluations were identified. The majority of evaluations (*n* = 10; 83.3%) involved cost effectiveness analysis. Only four studies (33.3%) included the cost of providing information about NIPT in their economic evaluation. Two studies considered the impact of test results on parents’ quality of life by allowing utility decrements for different outcomes. Some studies suggested that the challenges of valuing information prohibited their inclusion in an economic evaluation.

**Conclusion:**

Economic evaluations of NIPTs need to account for the costs and outcomes associated with information provision, otherwise estimates of cost effectiveness may prove inaccurate.

**Electronic supplementary material:**

The online version of this article (10.1007/s10198-019-01082-x) contains supplementary material, which is available to authorized users.

## Introduction

Prenatal testing involves the use of a range of tests to screen and diagnose abnormalities in pregnancy. Prenatal “screening” is usually used to determine the risk of delivering a baby with an abnormality [1]. Prenatal “diagnosis” techniques, such as amniocentesis and chorionic villus sampling (CVS), are then offered to confirm or reject a diagnosis of foetal abnormalities [1]. Conditions that can be detected vary from developmental abnormalities, such as neural tube defects, to genetic disorders [2]. Globally, one of the most common prenatal screening tests seeks to identify foetuses at risk of being born with Down’s syndrome (DS), but screening for other aneuploidies are often included as well [3]. Down’s syndrome is the most common congenital cause of mental disability caused by a complete or partial third copy of Chromosome 21 [4].

Prenatal screening technologies for DS and other aneuploidies have evolved significantly over the past several decades from determining risk, simply using maternal age, to the addition of serum protein markers and ultrasound scans to measure nuchal translucency (NT) [5]. Screening programmes vary between countries [6–8] but generally involve a first trimester triple or quadruple test comprising a combination of: two serum proteins, beta unit of human chorionic gonadotrophin (ß-hCG) and pregnancy-associated plasma protein A (PAPP-A) and NT measurement [5]. However, these existing screening technologies exhibit negative characteristics such as a moderate risk of false-positive results (2–7%) [9]. Furthermore, the diagnostic tests are invasive and pose the risk of complications to the mothers and their baby [10]. According to the UK National Health Service Foetal Anomaly Screening Programme, the risk of miscarriage with invasive testing is 1–2% [9, 11].

Non-invasive prenatal testing (NIPT) is a more recent test used to identify aneuploidies, which works by detecting cell-free foetal DNA in maternal plasma from a blood test [12]. Clinical laboratories assess the likelihood of foetal aneuploidy by analysing cell-free DNA in maternal serum using various techniques, including shotgun massively parallel sequencing (s-MPS), targeted massively parallel sequencing (t-MPS) and single nucleotide polymorphism (SNP)-based approaches [13].

NIPT has been suggested to be a clinically useful test; a systematic review and meta-analysis of clinical trials suggested the strategy to be highly accurate (99.7% sensitivity and almost 100% specificity) for the detection of DS in high to average risk populations [14]. NIPT may also be used for detecting other aneuploidies but with much lower precision [15]. NIPT cannot detect non-genetic abnormalities such as neural tube defects [13]. The high accuracy of NIPT is a key advantage because the number of invasive diagnostic tests could potentially decrease [10]. Furthermore, the test can be performed as early as at 10 weeks of pregnancy [10]. For this reason, professional societies such as the International Society for Prenatal Diagnosis and the American College of Obstetricians and Gynaecologists (ACOG) recommend NIPT as an advanced screening test for women at high risk for foetal aneuploidy [16, 17] and it is available in the private healthcare market.

The cost of NIPT varies greatly from several hundreds to thousands of US dollars and the high cost of testing may restrict its widespread use [10]. The relative cost effectiveness of NIPT is likely to inform whether it will be used widely, especially in publicly funded health care systems. This is because the provision of funding for new interventions removes funding allocated to the existing programmes. Economic evaluations can be used to provide evidence as to whether the health gained by the patients who receive the new intervention is greater than the health lost by those individuals who forgo interventions in the healthcare system [18]. A recent systematic review by Nshimyumukiza et al. (2018) identified economic evaluations of NIPT [19]. The results of this review found that universal NIPT was not cost effective for publicly funded screening (when future costs of raising a child with disability were not considered), unless there is a substantial decrease in the direct cost of NIPT. However, many economic evaluations of NIPT recognised that the estimated costs may not be accurate and the authors of the review recommended that the design of future studies could be improved to include all relevant health outcomes and costs for the mother and infant [20].

Such relevant costs and health outcomes should include the cost of information provision and the health impact resulting from the difficult choices faced by parents when receiving screening results [21]. In a prospective cohort study from the UK, approximately one-third of parents (31%) with a confirmed positive NIPT result chose to continue their pregnancy, indicating a potential need for additional information to prepare for having a child with Down’s syndrome and not necessarily for decision making about termination of pregnancy [15]. On the other hand, the majority of the women with a positive NIPT choose to terminate the pregnancy [22]; therefore, it is important to explain the uncertainty around false-positive and false-negative results to parents clearly.

Pre-test counselling alongside NIPT is recommended by several published guidelines [13]. Pre-test counselling should ensure that informed consent is gained from the parent which requires the provision of accurate information about screening, its advantages and limitations. The education of patients currently undergoing NIPT does not meet these standards for informed consent consistently. A survey of American women who had experienced NIPT found that most appeared satisfied with their understanding of NIPT and the testing process, yet they may not have appreciated the limitations of this screening method fully [13].

Alternatively, NIPT may help to reduce the anxiety that parents experience during the screening process. A questionnaire study conducted with Finnish women in 1998 found that women believed that serum screening was more associated with finding diseases or abnormalities than with the ultrasound, and that the main reason for screening was for reassurance. However, the sample was also found to have limited knowledge regarding the sensitivity of the screening tests and the risks associated with diagnostic tests [23]. Furthermore, as the uptake of NIPT increases, it is important that routine use does not hinder parental informed consent and autonomous choice as has been argued to occur in the provision of some newborn bloodspot screening programmes [13, 24].

A previous systematic review of economic evaluations of newborn bloodspot screening programmes and technologies found that few studies included the cost of providing information about testing or the health impact of not providing this information [21]. Only five studies included a cost of information provision, which while small for each patient (ranging from GBP £0.40 to EUR €5.41) would sum up to significant levels given the large number of babies that receive screening. Potential health disutilities arising from poor information provision occurred when parents received false-positive results and ranged from 0.0005 to 0.0125 quality-adjusted life years lost. While these values are relatively small, it is arguable that parents going through prenatal screening for aneuploidy are likely to experience larger levels of anxiety due to the potential consequences of the test.

The aim of this systematic review was to identify the extent to which economic evaluations of NIPT have accounted for the need to provide information for parents alongside testing and the associated costs and health outcomes of information provision.

## Methods

A systematic review was conducted which aimed to find all economic evaluations of NIPT. The review was reported according to published guidelines (PRISMA) [25].

### Literature search

The electronic databases Medline, Embase, PsycINFO, CINAHL were searched for relevant published economic evaluations using Ovid. The search strategy comprised key terms, such as “pregnant women” and “antenatal screening”, and a search filter to identify economic evaluations (see supplementary appendix 1). All searches were conducted in February 2018. Results were exported to Endnote reference manager and screened manually to remove duplicate records. Two systematic reviews of economic evaluations of NIPT were published when undertaking this study [19, 26]. The studies identified by these two systematic reviews were cross-checked with the studies identified by this systematic review to ensure that all published economic evaluations of NIPT were found.

### Inclusion and exclusion criteria

The identified economic evaluations, titles and abstracts were screened by two independent reviewers (NJ and CV/SG) to assess whether the study satisfied the criteria specified for inclusion in the review (see Table 1). All types of full economic evaluation (cost effectiveness; cost–utility and cost–benefit analyses) were eligible for inclusion in the review. Cost-minimisation studies and budget impact analyses were excluded.

**Table 1:** Inclusion and exclusion criteria

	Inclusion	Exclusion
Population	Pregnant women	Anyone other than pregnant women Studies on animals
Intervention	NIPT to detect foetal abnormalities	Antenatal screening or diagnostic interventions for sickle cell disease, thalassaemia, diabetes, syphilis, HIV, hepatitis Neonatal screening Pre-conception screening
Comparators	Other approaches to screening for foetal abnormalities including but not limited to, nuchal translucency, triple and quadruple testing	Antenatal screening or diagnostic interventions for sickle cell disease, thalassaemia, diabetes, syphilis, HIV, hepatitis Neonatal screening Pre-conception screening
Outcome	Cost effectiveness expressed in cost per unit of outcome (for example, cost per unit of clinical outcome, cost per QALY/DALY, cost per unit of monetary benefit)	Clinical outcomes only Cost outcomes only
Study type	Full economic evaluation (cost-effectiveness analysis; cost–utility analysis; cost–benefit analysis)	Anything other than a full economic evaluation, e.g., systematic reviews, conference abstracts Non-full text reports, cost-minimisation analyses and budget impact analyses
Other	Papers written in English language	Papers written in languages other than English
Economic model type	Decision-analytic models	Other types of models Clinical trials

### Data extraction and synthesis

Three reviewers (NJ/CV/SG) extracted the data from each identified study using a structured data collection form (see supplementary appendix 2) based on the Consolidated Health Economic Evaluation Reporting Standards (CHEERS) framework [27]. The data extracted incorporated the (1) author, year of publication and country in which the study was conducted, (2) intervention and comparator, (3) perspective and target population, (4) decision-analytic model type, time horizon and discount rate (5) resources used, cost sources, and price year (6) valuation of costs and benefits, (7) whether costs for the provision of information were considered, whether the impact of information on the parents’ outcomes was considered, the estimated uptake of NIPT, and (8) whether omitting the cost or outcome implications of information provision was recognised as a limitation. The estimated uptake of NIPT was extracted from included studies, as it has been hypothesised that there is, albeit complex, a relationship between information about screening and participation rates [28]. The results were tabulated and summarised as part of a narrative synthesis.

The methodological quality of the included studies was not assessed, as it was not believed that the quality of the included studies in terms of their validity as economic evaluations would be relevant for the primary question of this review: whether economic evaluations included the costs and outcomes associated with providing information to parents.

## Results

**Fig. 1:**
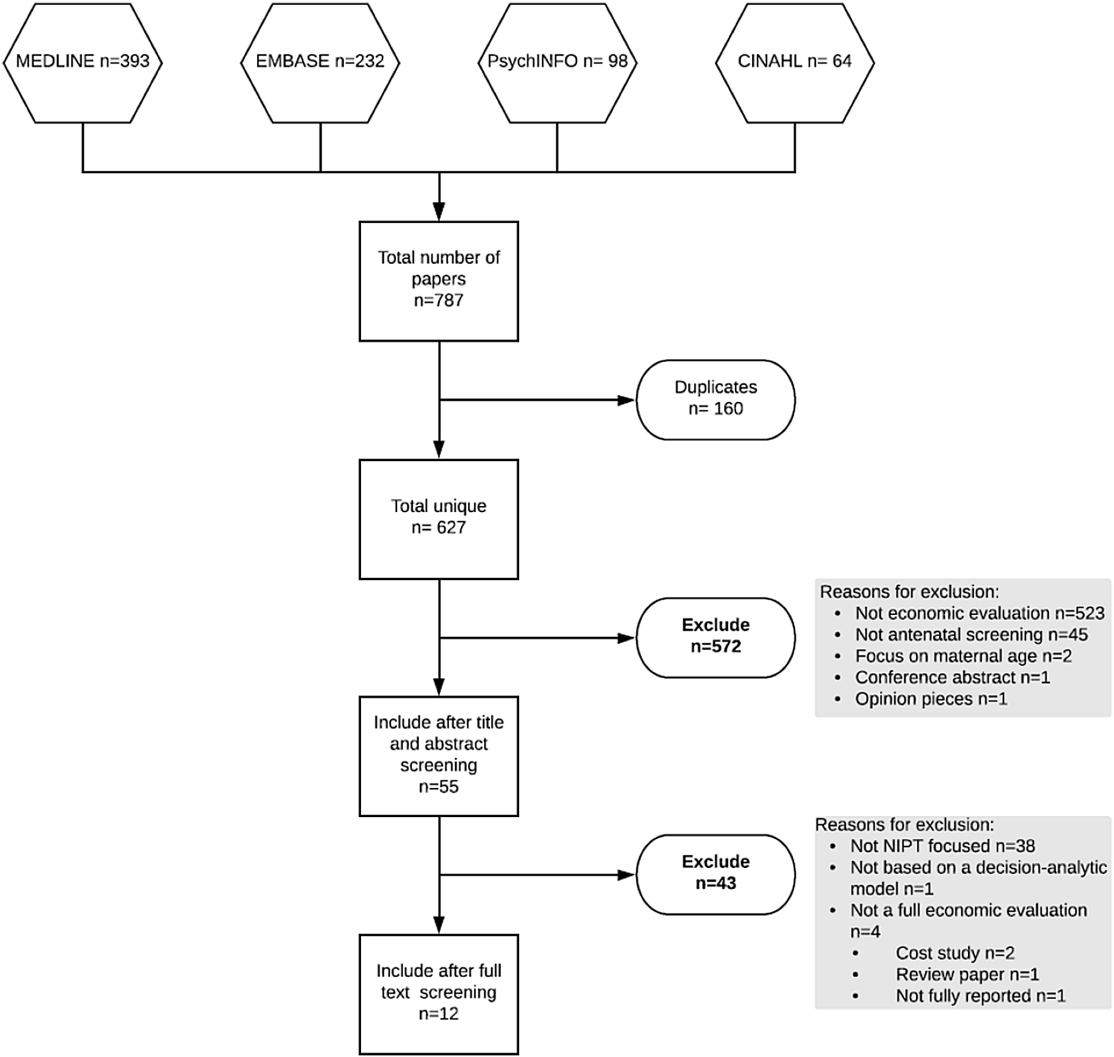
PRISMA flow diagram

Figure 1 summarises the study identification and inclusion process. A total of 627 unique articles were found through searching relevant databases. After applying the inclusion and exclusion criteria (see Table 1), a total of 12 economic evaluations were identified and included in this review (see Table 2 in supplementary appendix 2). 572 articles were removed in title and abstract screening. Of the 55 articles that remained, 17 were NIPT-focused articles. Five of these were unsuitable. The most common reason was that they were not full economic evaluations: Nshimyumkiza et al. [19] was a systematic review, Cuckle et al. [29] was a letter to the editor in response to an included study, Benn et al. [30] and Odibo and Garfield [31] reported cost studies only and the evaluation by Connor et al. [32] was not based on a decision-analytic model.

### Summary of included studies

The studies included in this review were published between 2013 and 2015. The majority of the included economic evaluations were conducted in the USA (*n* = 6; 50%). Other countries were: Australia (*n* = 2; 16.7%), the Netherlands (*n* = 1; 0.8%), UK (*n* = 1; 0.8%), Belgium (*n* = 1; 0.8%), and Canada (*n* = 1; 0.8%). The study population varied greatly among the 12 reviewed articles. Most articles modelled their evaluation on a theoretical cohort of women representative of the number of annual pregnancies from a previous year in their respective countries. Some articles specified their target population according to the stage or condition of pregnancy. For example, Ayres et al. [6] specified “singleton pregnancies” and Neyt et al. [33] specified “singleton pregnancies at gestational week 10”. Other papers also tended to specify pregnant women at an early stage; Okun et al. [34] specified women in the “first trimester”, and Song specified “12 weeks of gestation” [35]. Kaimal et al. [36] was unique in that the cohort of women already desired screening.

A wide range of comparisons of NIPT technologies were performed in the 12 economic evaluations. Eight studies compared an existing screening scheme [which usually involved first trimester maternal serum screen (MSS) blood tests and a nuchal translucency (NT) ultrasound scan followed by invasive diagnostic testing (IDT) if high risk] with contingent NIPT testing (adding NIPT as a second-line screening test before IDT) [3, 6, 7, 12, 33, 34, 37, 38]. Two studies placed conditions on the use of NIPT with age [6, 35]. Ayres et al. (2014) and Song et al. (2013) compared the use of first-line NIPT for women > 35 years old with NIPT as a contingent test for women < 35 [6, 35]. Ten studies compared scenarios with NIPT as first line and only screened followed by IDT if at high risk [3, 6–8, 16, 33–37]. Ohno et al. [16] looked at NIPT as the only screening and diagnostic tool and Cuckle et al. [37] evaluated a strategy where NIPT replaced IDT.

### Types of economic evaluations

The majority of economic evaluations were cost effectiveness analysis (CEA) (*n* = 10; 83.3%) [3, 6, 7, 12, 33–35, 37–39]; the remaining studies reported cost–utility analysis (*n* = 2; 16.7%) [16, 36]. The unit of outcome included in the CEA analysis was the number of DS detected (*n* = 8) [3, 6, 7, 33, 34, 36, 38, 39], the number of invasive diagnostic tests (IDT) performed (*n* = 5) [3, 16, 34–36], the number of procedure-related losses (PRL) (*n* = 5) [6, 12, 16, 34, 39], and the number of live births with DS (*n* = 2) [16, 36]. The perspectives taken in the economic evaluations were: only “payer” (*n* = 5, 42%) [7, 33, 34, 37, 39]; only “societal” (*n* = 1, 8%) [16]; and a combination of “societal”, “payer” and “governmental” (*n* = 1, 8%) [38]. The remaining studies did not report the target perspective (*n* = 5, 42%) [3, 6, 12, 35, 36].

### Cost of providing information to parents

Four studies [3, 6, 35, 38] included the cost of providing information about NIPT in their economic evaluation. Song et al. (2013), Ayres et al. (2015), and Fairbrother et al. (2016) included the cost of information provision by accounting for an additional visit with a doctor to discuss results, i.e., a positive NIPT result [3, 6]. In the study by Ayres et al. (2015), it was not clear which doctor the parents would see but costs ranged from AUS $36.30 to $60.00 (2014 prices) for a general practioner (GP) visit or AUS $47.15 to $200.00 for an obstetrician. Song et al. (2013) included the cost of an “office visit with counselling” to be US $120 (range: $40–$200, 2012 prices), but it was not clear what this cost was based on. This value was also used by Fairbrother et al. (2016), although a lower bound of US $80 was used; the cost was not updated to reflect current prices despite this study being published in 2016.

Walker et al. [38] accounted for the cost of genetic counselling alongside all comparator screening tests rather than just NIPT testing. This value of US $160 (2013 prices) with a range of $91–$247 was identified from the 2013 Medicare Physician Fee Schedule. No studies included a cost of providing information about NIPT before the screening was undertaken.

Two studies considered the cost of information provision in their sensitivity analysis [3, 38]. Fairbrother et al. [3] found that the cost effectiveness of NIPT was sensitive to the total cost of the intervention which included the cost of counselling. Walker et al. [38] did not report the impact of changes in the cost of counselling on the cost effectiveness of NIPT.

Of the studies that did not include a cost of information provision for NIPT, one study stated that pre-test counselling was included in the costs for invasive diagnostic testing, but the estimated cost of NIPT testing did not include this [37]. Of the eight other studies [7, 12, 16, 33–36, 39] that did not include costs of providing information, only two studies qualitatively recognised this as a limitation [33, 34]. Okun et al. (2014) suggested that administration of post-test counselling and follow-up is particularly important to evaluating the introduction of a new technology into an existing system; however, they were unable to capture this information, because NIPT was being delivered outside of the existing centrally monitored prenatal screening programmes [34].

### Impact of imperfect information on the parents’ quality of life

A lack of effective information provision alongside NIPT testing may have an impact on parents’ quality of life through increased anxiety. Two studies considered the impact of test results of differing levels of certainty on parents’ quality of life [16, 36]. Kaimal et al. (2015) conducted a time-trade-off exercise with 281 women in San Francisco, California to value the impact of different test results on the quality of life. Test-related utilities ranged from 0.931 for mothers receiving prenatal testing and receiving a low-risk result with no further testing required to 0.655 for mothers receiving high-risk results from screening, a confirmed positive result from diagnostic tests, and who decide to continue with the pregnancy. This disutility of 0.276 to the mother highlights the potential need for counselling alongside testing to support parents and mitigate the impact of anxiety. Furthermore, disutilities arise from equivocal results at the screening and diagnostic stages [36]. When a mother received no result from cell-free DNA screening followed by a normal result from a diagnostic test, a disutility of 0.003 was still experienced. When the mother decided not to receive diagnostic testing after receiving no result from the cell-free DNA screening, they only experienced a utility of 0.763, representing a disutility of 0.168 from full health.

Unlike in many examples used in the paper, for parents that received a high-risk result from multiple marker or cell-free DNA screening and then a “variant of unknown significance” result from diagnosis, a higher disutility was applied when deciding to terminate the pregnancy (0.762) than if they continued (0.806) [36]. This may indicate the significant anxiety felt from making the decision to terminate a pregnancy without certainty that a child would be born with an aneuploidy.

Ohno et al. [16] also included specific utility values for test-related outcomes. For a parent who received a false-positive result from screening and gave birth to a healthy baby, a utility of 0.96 was applied for 1 year following birth. For parents that received a false-negative result and then delivered a child with DS, a utility of 0.71 was applied for the rest of the mother’s life (an average of 55.4 years). While these utility values were based on assumptions by the authors, the potential for false-negative results to generate a loss of 5.54 undiscounted QALYs highlights the need to inform parents of the imperfect nature of tests.

Of the ten studies that did not consider the impact of NIPT on the parents’ health outcomes [3, 7, 12, 33–39], two studies reported this as a limitation [12, 37]. O’Leary et al. [12] stated that “NIPT has the potential to improve mothers’ experience of prenatal testing”, whereas Cuckle et al. [37] suggested that the impact on parents, such as minimising the distress associated with loss, were non-tangible benefits and could not be measured.

### The impact of uptake of NIPT

The provision of information about NIPT testing may also have an impact on the level of uptake for the service which may, in turn, have an impact on total costs and outcomes. In 2013, the average uptake reported by all articles was 75% [12, 16, 35, 37]; in 2014, the average uptake reported was 80% [6, 7, 33, 34, 39]; in 2015, the average uptake reported was 75.8% [3, 36, 38]. This may suggest that the anticipated uptake of NIPT testing has remained at a similar level throughout these years despite increasing evidence as to the benefits of testing.

The majority of estimates for uptake reported by economic evaluations were assumptions made by the authors. Ayres et al. (2014) and Beulen et al. (2014) stated that their values were based on assumptions without any references [6, 7]. Three studies [8, 12, 33] assumed that uptake would be same as the current uptake of invasive testing, whereas Fairbrother et al. [3] assumed the same degree of uptake as the first trimester screening. Three studies [34, 35, 38] based their estimates of uptake on what was written in other papers and non-published data. As the target population in the study by Kaimal et al. [36] was all women who desired prenatal testing, the uptake was assumed to be 100%. Finally, Cuckle et al. (2013) did not state where their estimate of uptake was derived from [37]. None of the included studies linked the uptake of NIPT testing to the provision of good quality information that helps parents to make informed decisions about screening.

## Discussion

This systematic review identified 12 economic evaluations of NIPT testing for aneuploidies. However, despite the need for informed consent, the difficult decisions that may have to be made by parents when receiving test results, and the inherent complexity of the testing process itself, only few of the evaluations have accounted for the need for, and impact of, information provision. Receiving positive or false-positive results from screening has been shown to significantly increase parental anxiety [40]. Providing information may come at a financial cost to the health service but is likely to provide benefits in reducing the anxiety that parents experience during screening for aneuploidies. Furthermore, it is possible that effective information provision may increase the uptake of a potentially cost effective intervention, thereby increasing the total health benefit to society.

Omitting the cost of information provision alongside NIPT screening may result in an overestimate of relative cost effectiveness of the intervention. Four (33%) of the studies in this review included a cost for information provision, which was commonly quantified as the cost of an additional visit to a clinician for counselling on the receipt of positive results. One study also considered the cost of counselling in their sensitivity analyses [3]. The costs of information provision ranged from AUS $36.30 to $200 in Australia to US $40 to $247 in the USA. While these values are small for an individual, the size of the population eligible to receive testing means that the total cost of information provision will be high. For example, WHO estimated that between 3000 and 5000 children are born with Down’s syndrome each year in the USA. Given the estimates for the cost of counselling in the USA, this could mean an additional cost between $120,000 and $1,235,000 per year. These figures are in addition to the cost of counselling provided to families who later decide to terminate the pregnancy. A study conducted in the state of Massachusetts in 2016 suggested that around 49% may decide to terminate the pregnancy, meaning that the cost of counselling could range from US $344,898 to $2,520,408 annually across the USA, although termination rates are likely to differ by state [41]. These approximations also omit counselling for parents with positive results for other aneuploidies such as Edward’s syndrome and Patau’s syndrome.

While the cost of counselling on receipt of NIPT results may be a significant omission from many economic evaluations, a more serious issue may be the omission of costs related to information provision prior to the screening process to all expectant parents. Such information is critical in allowing parents to make an informed decision about whether or not to take part in screening for aneuploidies and in preparing parents for the potentially difficult decisions that may arise from screening. None of the studies identified in this review included such a cost. While the cost of providing information about NIPT screening to individual sets of parents may be small, all expectant parents who have access to screening should have access to this information.

A previous systematic review of the inclusion of information provision in economic evaluations of newborn screening programmes found that this cost could be as high as €5.41 (2002 prices, approximately US $7.04 in 2018 prices) and a micro-costing study conducted in the UK estimated that the expected cost per set of parents could be £17.65 (2014 prices, US $24.81 in 2018 prices) [21, 24, 42]. It is arguable that the decisions resulting from positive screening results from NIPT testing, terminating or continuing a pregnancy, may be more difficult than those arising from NBS screening and that more information may be required for the parent(s) to make informed decisions. Therefore, by omitting the provision of such information, the cost of NIPT may be underestimated.

Failing to provide effective information about NIPT screening may result in higher anxiety for the parent(s) when receiving test results. No studies linked the provision of information for NIPT testing to levels of parental quality of life. The low number of studies accounting for the impact of test results on parental HRQoL is likely a result of the use of cost effectiveness analysis in the majority of these studies which measure outcomes in natural units.

Two studies did include disutilities for parents that received distressing results. Kaimal et al. [36] conducted a time-trade-off study to elicit utility values for different health states pertaining to the receipt of test results. These values highlighted the significant impact that results can have on parents and the implications of uncertain results arising during the screening and diagnostic process. Similarly, Ohno et al. [16] included disutilities for false-positive and false-negative results with the latter assumed to have a significant lifelong impact on the parents.

However, by including such utility values for both the intervention and comparator, the relative cost effectiveness of NIPT, which reduces the risks of uncertain results, may improve. The cost effectiveness of NIPT could also be affected by the perceived accuracy of the test which could mean that parents make decisions about the pregnancy without receiving invasive but confirmatory diagnostic tests. Kaimal et al. [36] raised the prospect of NIPT testing being used after multiple marker testing as a diagnostic itself and utility values for the outcomes associated with this strategy were higher than the more reliable but riskier invasive diagnostic testing strategy. If NIPT screening is deemed to be a cost effective use of resources, then failing to provide adequate information about testing may lead to a sub-optimal uptake of the intervention and missed potential societal health gains. While some studies in this review included uptake in their economic models, none linked this to the quality of information provision as part of the screening programme. Alternative methodological approaches such as discrete choice experiments may be useful to determine what information parents would like to receive about NIPT screening and how they would like this to be delivered [43, 44].

There may be challenges in quantifying utility, particularly when it is related to non-health costs or benefits, for example, the intrinsic “value of knowing” associated with a true test result [45]. It might therefore be useful for economic analyses to include data derived from alternative valuation methods such as discrete choice experiments (DCEs) or contingent valuation studies. DCEs have been used to understand the preferences of women and healthcare professionals for NIPT and understand how they balance the associated benefits and risks. DCE data have also been used to predict the probability of a participant choosing NIPT in different scenarios. As studies in this systematic review often assumed participation rates, the results of a DCE could be used in a model-based economic evaluation for both utility and uptake parameters.

This systematic review had some limitations. Only decision-analytic models and not trials were included in this review. It is therefore possible that some trials of NIPT testing which included the cost of information provision and its impact were excluded from the review. However, the authors are only aware of one such trial and the complex nature of NIPT testing mean that decision-analytic models are a more practical vehicle to compare all relevant testing strategies in a cost effectiveness analysis [29]. This review also only looked at information provision alongside NIPT testing; expanding the review to all economic evaluations of screening for aneuploidies may have identified cost and disutility estimates for information provision alongside other technologies which may have been useful for researchers seeking to include such input parameters in future economic evaluations.

Including these additional costs and outcomes in economic evaluations of NIPT testing is critical to gain an accurate estimate of the cost effectiveness of this clinically useful but costly intervention. This is particularly important when screening for aneuploidies, as information provision is essential to ensure that parents make an informed choice about screening. However, few economic evaluations identified in this systematic review accounted for such costs and outcomes which may lead to inaccurate estimates of the cost effectiveness of NIPT. These findings may be symptomatic of a wider failure in health economics to account for such service delivery costs [46].

## Electronic supplementary material

Below is the link to the electronic supplementary material.

Supplementary material 1 (DOCX 19 kb)

Supplementary material 2 (DOCX 25 kb)
