# Supplementary material for: The role of information provision in economic evaluations of non-invasive prenatal testing: a systematic review
Source: Eur J Health Econ. 2019 Jun 22;20(8):1123–31. doi: 10.1007/s10198-019-01082-x (PMC6803567; doi:10.1007/s10198-019-01082-x)
Supplement: Supplementary file 1 — Supplementary material 1 (DOCX 19 kb) [file 10198_2019_1082_MOESM1_ESM.docx]

Supplementary Appendix 1: Search strategy

| Economic evaluations NHS EED**NHS EED MEDLINE using OvidSP** 1     Economics/  2     exp "costs and cost analysis"/  3     Economics, Dental/  4     exp economics, hospital/  5     Economics, Medical/  6     Economics, Nursing/  7     Economics, Pharmaceutical/  8     (economic$ or cost or costs or costly or costing or price or prices or pricing or pharmacoeconomic$).ti,ab.  9     (expenditure$ not energy).ti,ab.  10     value for money.ti,ab.  11     budget$.ti,ab.  12     or/1-11  13     ((energy or oxygen) adj cost).ti,ab.  14     (metabolic adj cost).ti,ab.  15     ((energy or oxygen) adj expenditure).ti,ab.  16     or/13-15  17     12 not 16  18     letter.pt.  19     editorial.pt.  20     historical article.pt.  21     or/18-20  22     17 not 21  23     exp animals/ not humans/  24     22 not 23  25     bmj.jn.  26     "cochrane database of systematic reviews".jn.  27     health technology assessment winchester england.jn.  28     or/25-27  29     24 not 28  30     limit 29 to yr="2010 -Current" **NHS EED EMBASE using OvidSP** - [**download as PDF**](http://www.crd.york.ac.uk/crdweb/PDFs/NHS%20EED%20EMBASE%2002122013.pdf) 1. Health Economics/ 2. exp Economic Evaluation/ 3. exp Health Care Cost/ 4. pharmacoeconomics/ 5. 1 or 2 or 3 or 4 6. (econom$ or cost or costs or costly or costing or price or prices or pricing or pharmacoeconomic$).ti,ab. 7. (expenditure$ not energy).ti,ab. 8. (value adj2 money).ti,ab. 9. budget$.ti,ab. 10. 6 or 7 or 8 or 9 11. 5 or 10 12. letter.pt. 13. editorial.pt. 14. note.pt. 15. 12 or 13 or 14 16. 11 not 15 17. (metabolic adj cost).ti,ab. 18. ((energy or oxygen) adj cost).ti,ab. 19. ((energy or oxygen) adj expenditure).ti,ab. 20. 17 or 18 or 19 21. 16 not 20 22. animal/ 23. exp animal experiment/ 24. nonhuman/ 25. (rat or rats or mouse or mice or hamster or hamsters or animal or animals or dog or dogs or cat or cats or bovine or sheep).ti,ab,sh. 26. 22 or 23 or 24 or 25 27. exp human/ 28. human experiment/ 29. 27 or 28 30. 26 not (26 and 29) 31. 21 not 30 32. 0959-8146.is. 33. (1469-493X or 1366-5278).is. 34. 1756-1833.en. 35. 32 or 33 or 34 36. 31 not 35 37. conference abstract.pt. 38. 36 not 37 39. limit 38 to yr="2010 -Current" **NHS EED PsycINFO using OvidSP** 1. "costs and cost analysis"/ 2. "Cost Containment"/ 3. (economic adj2 evaluation$).ti,ab. 4. (economic adj2 analy$).ti,ab. 5. (economic adj2 (study or studies)).ti,ab. 6. (cost adj2 evaluation$).ti,ab. 7. (cost adj2 analy$).ti,ab. 8. (cost adj2 (study or studies)).ti,ab. 9. (cost adj2 effective$).ti,ab. 10. (cost adj2 benefit$).ti,ab. 11. (cost adj2 utili$).ti,ab. 12. (cost adj2 minimi$).ti,ab. 13. (cost adj2 consequence$).ti,ab. 14. (cost adj2 comparison$).ti,ab. 15. (cost adj2 identificat$).ti,ab. 16. (pharmacoeconomic$ or pharmaco-economic$).ti,ab. 17. or/1-16 18. (task adj2 cost$).ti,ab,id. 19. (switch$ adj2 cost$).ti,ab,id. 20. (metabolic adj cost).ti,ab,id. 21. ((energy or oxygen) adj cost).ti,ab,id. 22. ((energy or oxygen) adj expenditure).ti,ab,id. 23. or/18-22 24. (animal or animals or rat or rats or mouse or mice or hamster or hamsters or dog or dogs or cat or cats or bovine or sheep or ovine or pig or pigs).ab,ti,id,de. 25. editorial.dt. 26. letter.dt. 27. dissertation abstract.pt. 28. or/24-27 29. (0003-4819 or 0003-9926 or 0959-8146 or 0098-7484 or 0140-6736 or 0028-4793 or 1469-493X).is. 30. 17 not (23 or 28 or 29) 31. limit 30 to yr="2010 -Current" **NHS EED CINAHL using EBSCO** S1        MH "Economics+" S2        MH "Financial Management+" S3        MH "Financial Support+" S4        MH "Financing, Organized+" S5        MH "Business+" S6        S2 OR S3 or S4 OR S5 S7        S1 NOT S6 S8        MH "Health Resource Allocation" S9        MH "Health Resource Utilization" S10      S8 OR S9 S11      S7 OR S10 S12      TI (cost or costs or economic* or pharmacoeconomic* or price* or pricing*) OR AB (cost or costs or economic* or pharmacoeconomic* or price* or pricing*) S13      S11 OR S12 S14      PT editorial  S15      PT letter  S16      PT commentary  S17      S14 or S15 or S16  S18      S13 NOT S17  S19      MH "Animal Studies"  S20      (ZT "doctoral dissertation") or (ZT "masters thesis") S21      S18 NOT (S19 OR S20) S22      PY 2009- S23      S21 AND S22 |
| --- |
