# Supplementary material for: The role of information provision in economic evaluations of non-invasive prenatal testing: a systematic review
Source: Eur J Health Econ. 2019 Jun 22;20(8):1123–31. doi: 10.1007/s10198-019-01082-x (PMC6803567; doi:10.1007/s10198-019-01082-x)
Supplement: Supplementary file 2 — Supplementary material 2 (DOCX 25 kb) [file 10198_2019_1082_MOESM2_ESM.docx]

***Data extraction table***

Table 2 Data extraction of 12 included articles

| Author, Year, country | Intervention & comparator | Study viewpoint and population | Evaluation vehicle (type)  Time horizon  Discount rate | Resource Use & Price Year (currency)  Cost of testing (range) | Valuation of Benefits | Were costs related to information provision included?  Was the Impact on patient considered?  What was the uptake and what was the range used? (%) | If costs or impact on patient not considered, has this been qualitatively identified as a limitation? |
| --- | --- | --- | --- | --- | --- | --- | --- |
| Ayres, (2014)  Australia | (1) Combined first trimester screening (CFTS) for all between 11-13 weeks gestation & IDT for high risk women, i.e. conventional Australian DS screening; (2) NIPT offered initially to high risk women (ie women over 35 years), CFTS offered initially for low risk mothers, NIPT subsequently offered for high-risk women who had CFTS initially, & IDT if NIPT is positive; (3) NIPT if a)older than 35 years, otherwise CFTS & NIPT for high-risk women, & IDT if NIPT is positive; (4) NIPT for all as primary screening & IDT if NIPT is positive. | Viewpoint:  Not reported  Study Population: Virtual cohort of 300,000 Australian singleton pregnant women | Decision analytic model  Time Horizon:  Not reported  Discount Rate:  Not reported | Resources:  Direct cost of CFTS, IDT, NIPT  Cost Sources:  MBS (low cost) or from a selection of private pathology and imaging pricing schedules (high cost) from health providers in South-East Queensland  Price year:  2014 Australian $  Total cost of testing range :  ($575-$900) | CEA:  Natural units:  (1) Number of DS detected and  (2) procedure related losses (PRL) avoided. | Information costs:  Yes, “NIPT-positive” women had an additional visit with their doctor to discuss results”  Impact on patient considered?  Yes, psychological impact of false positives considered.  Uptake of NIPT range:  100%  “Assumption” | -  - |
| Beulen, (2014)  The Netherlands | (1) First trimester combined testing (FCT) initially then IDT if high risk (conventional screening in Netherlands) (2) FCT for all, NIPT as optional secondary screening test for those at high risk, IDT for high-risk women;  (3) NIPT for all as primary screening, IDT for high-risk women. | Viewpoint:  Health care perspective  Study population: Cohort of 180,000 pregnant women (annual pregnancies in the Netherlands) | Decision analytic model  Time horizon:  From 10 weeks gestation up to birth  Discount Rate:  Not reported | Resources:  Direct costs of screening and diagnostics, i.e. PCT, second trimester anomaly scan, invasive diagnostic testing, array analysis, NIPT  Cost Sources:  Dutch Healthcare authority, national university medical centres, health insurance companies  Price year:  2013 Euro  Average total cost of testing:  775.82 Euros | CEA:  Natural units:    (1) Trisomy 21 diagnosed;  (2) Live birth with trisomy 21 avoided; (3) Iatrogenic miscarriage avoided. | Information costs:  No  Impact on patient considered?  No  Uptake of NIPT range:  Different depending on age:  Less than or equal to 35: 60%  Greater than or equal to 35: 70%  “Assumptions were made after the introduction of NIPT in Dutch health care, based on studies evaluating women’s expressed interest and recent publications on actual choice made following the implementation of NIPT in prenatal care” reference the paper | No  No |
| Cuckle, (2013)  USA | (1) NIPT for all replacing all current screening (combined and quadruple tests), then IDT offered for diagnosis; (2)Conventional screening & NIPT for 10-20% of women with highest risks then IDT if positive  (3) Conventional screening and NIPT (replacing IDT) if positive. | View point:  Public health purchasers  Study population: Not reported | Decision analytic model  Time horizon:  Not reported  Discount Rate:  Not reported | Resources:  Pre and post procedure genetic counselling, ultrasound procedure, laboratory costs  Cost Sources:  Assumed values based on costs observed in USA and Europe  Price year:  Not reported (US dollar, year unknown)  Average total cost of testing:  $1000-1500 | CEA:  Natural units:  (1) Case of Down’s syndrome births avoided | Costs:  No  Although it does state that cost of invasive prenatal diagnosis was assumed to include pre-procuedure and post procedure, genetical counselling” Current costs of NIPT does not include these, but in the future this should be taken into consideration  Impact on patient considered?:  No  Uptake of NIPT range:  In the baseline case the uptake of the screening was assumed to be the same for all modalities : 100%  In the variant modalities the uptake rates were varied to 95%, 90%, 85%, 80%  The sources of these figures not stated, but it is said that “it is possible that the uptake of universal and contingent cfDNA testing will be higher than for conventional screening, because of the reduced need for invasive prenatal diagnosis” | Justification and limitation of only including costs of pre-test counselling and not post- test counselling mentioned  Yes “There are substantial non-tangible benefits in minimizing distress associated with losses” |
| Fairbrother, (2015)  USA | (1) NIPT for all in first trimester, IDT if high risk;  (2) First trimester combined screening (FTS) with serum markers and ultrasound, IDT if high risk | View point:  Not reported  Study population: General pregnancy population (4 million US women) | Decision analytic model  Time horizon:  Not reported  Discount Rate:  Not reported | Resources:  Costs associated with screening tests, invasive testing, office visits and counselling, termination procedures, cost of each trisomy birth.  Cost Sources:  Medicare 2014 fee Schedule , Bureau of labor statistics  Price year:  2014 USD  Total cost of testing range:  $400-700 | CEA  Natural Units:  (1) Number of trisomy cases identified;  (2) Reduced number of invasive tests performed  (3) Reduced iatrogenic foetal loss | Costs:  Yes  “All screen positive tests were assumed to have follow-up counselling, which generated one additional office visit”.  Impact on patient considered?:  Yes, impact regarding convenience considered, i.e. specialist referral for NT ultrasound in FTS can be inconvenient for patients, whereas NIPT allsof for all pregnant women to have equal access.  Uptake of NIPT range:  70%-85%  They chose 70% as it is same uptake as current FTS, but the increments up to 85% was for sensitivity analysis | -  No further comments on psychological impacts for patients mentioned. |
| Kaimal, (2015)  USA | (1)Chromosomal microarray  (2) Multi marker screening  (3) NIPT  (4) NT alone  (5) NT in combination  (6) NT in sequence  (1)MMS (first and second trimester serum analysis and NT measurement), IDT if additional information desired after screening  (2)MMS with secondary screen with NIPT or diagnostic testing if additional information desired  (3)NIPT as the only and primary screening, diagnostic testing if additional information desired  (4) Concurrent NIPT and NT measurement as primary and only screen, with diagnostic testing if additional information required  (5) Concurrent NIPT and MMS as primary screen , with diagnostic testing if additional information required  (6)diagnostic testing without prior screening | View point:  Not reported  Study population:  Theoretical cohort of 100,000 pregnant women who desire prenatal testing | Decision analytic model  Time horizon:  Lifetime  Discount rate :  3% | Resources:  Serum portion of integrated screen, nuchal translucency ultrasound test, amniocentesis with chromosomal microarray analysis, NIPT  Cost Sources:  Literature and clinical resources  Price year:  2014 US $  Total cost of testing range :  $1796 | CUA  QALY  (1) Prenatal detection or birth of neonate with a significant chromosomal abnormality  (2) Diagnostic procedures performed  (3) Maternal QALY  (4) Costs | Costs:  No  Impact on patient considered?:  Not explicitly, although :  False positives considered in QALY  Uptake of NIPT range:  100%  “because the theoretical cohort that only includes women desiring prenatal testing” | No  No, it does not mention the emotional impact on women, just societal and financial implications  “it is impossible to capture the full complexity of individual patient-provider decision-making as well as societal and financial implications in a model-based approach” |
| Morris, (2014)  UK | (1) NIPT as contingent testing: DS CFTS screening, if high risk NIPT is offered, if NIPT positive then IDS offered (2) NIPT as first line screening for DS, if NIPT positive then IDS is offered. | View point:  UK National Health Service  Study population:  10,000 pregnant women who are representative of the screening population in England | Decision analytic model, decision planning tool (DPT)  Time horizon:  Short time (from initial screen to final diagnosis)  Discount rate:  1% | Resources:  Combined test in first trimester, quadruple test in second semester, cost of repeat nuchal translucency measurements, NIPT, invasive tests (Staffing, administration, equipment, overheads), costs of delivering the screening test results (low risk results via 2nd class mail and high risk results via telephone.).  Cost Sources:  Pre-existing validated cost model of DS screening in the NHS  Price year:  UK 2011-2012 £  Total cost of testing range :  (£50-750) | CEA  Natural units:  (1)Number of DS cases detected;  (2) number of procedure related miscarriages;  (3) Spontaneous foetal loss | Costs:  No  Impact on patient considered?:  No  Uptake of NIPT range:  Different uptake for  Affected preganacies:90%  Unaffected pregnancies:80%  “Assumed that the base case uptake of NIPT would be same as for invasive testing in the current DS programme” | No  No |
| Neyt, (2014)  Belgium | (1)Current practice in Belgium: CFTS, IDS if high risk  (2)NIPT as a contingent test: CFTS, NIPT if high risk, IDT if NIPT positive  (3)NIPT as primary first line screening test, IDT if NIPT positive | View point:  Payer  Study population:  129,199 singleton pregnancies at gestational week 10 | Decision analytic model  Time horizon?  From 10 weeks’ gestation up to birth)  Discount rate:  0 | Resources:  screening, adverse effects, pregnancy termination  Cost Sources: National Institute for Health and Disability insurance (NIHDI)  Price year:  Euro 2013  Total cost of testing range :  ($460)kept at the same price as current test | CEA  Natural units:  (1)Number of T21 diagnosed | Costs:  No; “No additional cost for NIPT counselling is included since it assumed that this would happen in a similar way as in the current screening approach and does not occur as an incremental cost”  Impact on patient considered?:  No  Uptake of NIPT range:  80-90%  Figure that is the proportion of women who have invasive test after positive screening | Yes “quality of life is of major importance”, however “the impact on life years are not clear enough to make proper calculations with a long-term horizon”  No |
| Ohno, (2013)  USA | (1) NIPT as a diagnostic tool: NIPT as the only screening and diagnostic tool  (2) NIPT as a screening tool: NIPT as the first and only screening tool, IDT if NIPT positive | View point:  Social perspective  Study population:  Theoretical cohort of high-risk pregnant women presenting for prenatal care at a gestational age early enough for first trimester screening | Decision analytic model  Time horizon:  Not reported  Discount rate:  3% | Resources:  NIPT, amniocentesis, elective termination, spontaneous abortion, raising a DS  Cost Sources:  Medical care component of the consumer price index  Price year:  2012 US dollars  Total cost of testing range :  ($0-5000) | CUA:  QALY  (1) DS babies born  (2) Amniocentesis  (3) Terminations with DS  (4) Terminations without DS  (5) PRL  (6) Spontaneous abortions | Costs:  No  Impact on patient considered?:  Yes, maternal QALYs for false-positive diagnosis and false negative diagnosis considered  Uptake of NIPT range:  100% ( QALY changed with different results. For false negative=number, false positive = number)  “The purpose was to model an extreme example” | - |
| Okun, (2014)  Canada | (1) Current programme (primary screen with combination serum markers and ultrasound markers), if high risk IDT offered.  (2) nuchal translucency as primary screen, if high risk IDT offered.  (3) First trimester screening is NIPT only, IDT offered inf NIPT positive  (4) Contingent NIPT (current programme, NIPT if high risk, IDT if NIPT positive)  a) with current FTS performance  b)Contingent NIPT at a fixed price: to result in overall cost neutrality  c)Contingent NIPT: with an improved detection rate  d)Contingent NIPT: with higher uptake of FTS  e)Contingent NIPT: with optimised FTS | View point:  Payer  Study population:  144,570 Ontario pregnant women | Decision analytic model  Time horizon:  Not reported  Discount rate:  Not reported | Resources:  NIPT, amniocentesis  Cost Sources:  Ariosa diagnostics, Gamma Dynacare Laboratories??  Price year:  Canadian $ 2012-2013  Total cost of testing range :  ($600-795) | CEA:  Natural units:  (1)Amniocentesis performed;  (2) Cases of DS detected prenatally  (3) Amniocentesis related losses of non-DS affected pregnancies | Costs:  No  Impact on patient considered?:  No  screening up take considered that the safety of a screening test was important  Uptake of NIPT range:  67-80%  Lower limit is from unpublished dtat from the Better Outcomes Registry and Network of Ontario, and upper limit is arbitrary | Yes  “ that administration of post-test counselling and follow up are particularly importat in evaluating the introduction of a new technology into an existing system, however since NIPT is currently delivered outside the centrally monitored existing prenatal programmes, they were unable to capture this information”  No |
| O’Leary (2013)  Australia | (1)Current practice in Australia (CFTS, IDT if high risk)  (2)CFTS, NIPT if high risk, IDT if NIPT positive | View point:  Not reported  Study population: 32,478 singleton pregnancies similar to that screened between Jan 2005 and Dec 2006 | Decision analytic model  Time horizon:  Not reported  Discount rate:  Not reported | Resources:  First trimester screening (blood test, ultrasound), second trimester screening, amniocentesis (procedure, karyotyping), chorionic villus sampling (procedure, karyotyping), NIPT  Cost Sources:  Australian Medicare rebates  Price year:  Australian dollar 2013  Total cost of testing range :  ($743) kept at the same price as current test | CEA:  Natural units:  (1) Cost per averted procedure-related loss | Costs:  No  Impact on patient considered?:  No  Uptake of NIPT range:  75.3%- 100%  “consistent with the uptake of invasive testing in the cohort” | No  Yes, “mentioned how women may choose to not have NIPT because it does not identify other karyotypes apart from DS”. Also mentions “ NIPT has the potential to improve mother’ experience of prenatal testing” |
| Song, (2013)  USA | (1)FTS (combined test only), iNT if high risk  (2) FTS (combined and quad test), iNT if high risk  (3)NIPT as first line screening for women over 35, or second line for women under 35 with high risk on FTS, INT if high risk | View point:  Not reported  Study population:  High risk patients (women >35 years with a positive conventional screening test). Modelling based on 4 million pregnant women cohort in USA | Decision analytic model  Time horizon:  Not reported  Discount rate:  3% | Resources:  Serum markers pregnancy associated plasma Protein A (papp-a), and beta-hCG, first trimester ultrasound (including NT), FTS, Quad screening of serum markers (AFP,estriol, hCG, Inhibin A), NIPT  Cost Sources:  Medicare 2012 Fee Schedule, Ariosa diagnostics)  Price year:  2012 US dollars  Total cost of testing range :  ($695-995) | CEA  Natural unit:  (1)Number of T21 cases detected  (2)Number of invasive procedures performed  (3)Euploid foetal losses | Costs:  No  Impact on patient considered?:  No  Uptake of NIPT range:  Proportion Advanced maternal age (AMA) (50-90%  Proportion with screen positive with FTS or INT that undergo screening with NIPT (95-100%)  “data on file and from other papers” | No  No  Impact for NIPT not directly considered, but maternal anxiety due to high false positives in conventional screening (FTS, INT) was considered |
| Walker, (2015) USA | (1)Conventional maternal serum screening, IDT if MSS high risk  (2) Universal NIPT: NIPT as primary screen, IDT if NIPT positive  (3) Contingent NIPT: MSS as primary screen, NIPT as secondary screen if high risk | View point:  Societal, Government and payer  Study population: 1,000,000 US pregnant women at 12 weeks of pregnancy | Decision analytic model using Microsimulation  Time horizon?  Lifetime  Discount rate 3% | Resources:  Screening, diagnosis and termination of pregnancy, life time costs associated with trisomy 21, 18 and 13  Cost Sources:  Literature, 2013 Medicare Physician fee schedule (MPFS), NIFTY  Price year:  US 2013 $  Total cost of testing range :  ($229-619) | CEA  Natural unit:  (1) cost per DS case detected. | Costs:  Yes  Cost of genetic councelling is considered  Impact on patient considered?:  No  (note: not on mothers, but “indirect costs” with raising child with DS considered” in societal persoective  Uptake of NIPT range:  (69-82.5%)  Assumptions based on other studies | -  No |
